# In Vitro and in Cellulo Sensing of Transition Metals Using Time-Resolved Fluorescence Spectroscopy and Microscopy

**DOI:** 10.1007/s10895-018-2335-z

**Published:** 2018-12-26

**Authors:** Robert Pal, Abigail C. J. Barker, Daniel Hummel, Lars-Olof Pålsson

**Affiliations:** Department of Chemistry, Durham University, Lower Mountjoy, Stockton Road, Durham, DH1 3LE UK

**Keywords:** Metal sensing, Fluorescence spectroscopy, Time-resolved fluorescence microscopy

## Abstract

In this work we demonstrate that time domain techniques can be used successfully to monitor realtively weak modulations of the fluorescence in sensing applications. The metal sensing complex Newport Green DCF™ can detect selected transition metals in vivo as well as in vitro. Incremental addition of Ni and/or Zn (in vitro) lead to a substantial reduction in the yield of the fast component in a bi-exponential fluorescence decay (*τ*_1_ = 150–250 ps) from 60% to 30–35%. This is rationalised as an inhibition of intra-molecular electron transfer in the NPG sensing complex due to metal complexation. In order to explore this effect in cellulo, NIH 3 T3 mouse skin fibroplast cells were pre-incubated with set levels of Ni and Zn, at a constant concentration of NPG. The fluorescence modulation in cellullo was subsequently studied employing both time-resolved fluorescence microscopy and confocal fluorescence microscopy. In correlation with the in vitro observations, similar effects were observed on the fluorescence decay in cellulo.

**Electronic supplementary material:**

The online version of this article (10.1007/s10895-018-2335-z) contains supplementary material, which is available to authorized users.

## Introduction

The challenge in sensing pH, pM or pX in biological systems lies in designing reliable systems for selective and sensitive detection of the target analyte and the subsequent signal transduction process. For in vitro measurements, sensing is readily achieved using a number of different approaches, such as optical spectroscopy and electrochemistry techniques [1–6]. Fluorescence spectroscopy in particular, is an excellent tool in this context due to its flexibility and inherent sensitivity. Modulation of fluorescence can be caused by a number of external factors such as the di-electric medium, electron and/or energy transfer and medium viscosity. In this work we show that modulations in the time-resolved fluorescence can be used to monitor even weak interactions between target analytes and a fluorescent metal sensing complex.

Related to the impact on the kinetics of the fluorescence emission is the modulation of the quantum yield (QY). Stern-Volmer quenching of the fluorescence, or inherently the (QY), is therefore a frequently used method that provides information about inter-molecular interactions and equilibrium constants between the analyte and the functionalised optical probe [7–9].

While sensing of bio-assays using fluorescence spectroscopy can produce very important information about the physiological environment, it is highly desirable to pursue sensing in bio-active systems such as living cells. This can in principle be achieved using confocal fluorescence microscopy leading to in vivo sensing and monitoring. In this pursuit it is problematic to monitor intensity variations from modulation of the QY and as the cell is an inhomogeneous system in contrast to bio-fluids. Cell-to-cell variations of uptake and probe concentration and accumulation, will substantially contribute to the observed error on the outcome of fluorescence intensity maps in confocal microscopy. Ratiometric optical probes have been developed to overcome this problem such as functionalised lanthanide complexes [9–11]. In this class of materials the optical probes have at least one distinct internal reference signal which therefore facilitates interpretation of the data without dependency on intra-cellular probe concentrations and/or fluorophore densities. However, generally fluorescence spectra of purely organic chromophores and transition metal complexes are broad and devoid of distinctive features that could be used as reference points in ratiometric sensing.

Furthermore, there are few commercial off-the–shelf optical probes for sensing with internal reference signal and this calls for the need of other methodologies to be exploited.

A way forward is to monitor the time-resolved fluorescence of the applied optical probes and this can now also be achieved in confocal fluorescence microscopy [1, 12–14]. The basic principle is to monitor the fluorescence lifetime which given by;

1 $$ {\tau}_{\mathrm{f}}={\left({k}_{\mathrm{f}}+\sum {k}_{\mathrm{nr}}\right)}^{-1} $$

where *k*_f_ is the radiative rate coefficient and ∑*k*_nr_is the sum of non-radiative decay processes. A modulation of the fluorescence lifetime has a direct effect on the QY which in turn is given by;

2 $$ {\phi}_{\mathrm{f}}=\frac{k_{\mathrm{f}}}{k_{\mathrm{f}}+\sum {k}_{\mathrm{nr}}} $$

Hence, an increase of ∑*k*_nr_ due to an intermolecular interaction will affect the QY accordingly and the fluorescence lifetime which is the experimentally accessible parameter, independent of concentration which can be a significant advantage over intensity based sensing.

In this present work, the concept of microscopic sensing using time-resolved fluorescence microscopy is applied to an important question in biology; namely sensing, determining and monitoring the localised *in cellulo* presence of metals. Metals play a vital role in bio-active systems, such as co-factors in enzymatic reactions, signalling processes, protein folding and general regulation of natural cellular homeostatic activity [15–21]. However, elevated or abnormal intracellular metal concentrations could lead to adverse effects on cellular function and health [19]. Zn is one of the most important metals in biological systems with labile Zn (Zn^2+^) concentrations in the typical range of 10^−12^ – 10^−9^ M in mammalian cells [22–24]. Another very similar transition metal, Ni, is naturally abundant in photosynthetic cells but considered to have toxic effects in mammalian cells and regarded as carcinogenic [25, 26]. For sensing of these two important transition metals optical probes based on fluorescein derivatives have been developed in recent years [23, 27–30]. The sensing moiety in these fluorescein based complexes as well as other similar systems is an electron exchanging group which, upon photo excitation of the signalling chromophore, exhibit a modulation of the fluorescence due to Photo induced Electron Transfer (PET) [31, 32]. This inherently affects the fluorescence intensity and QY, but crucially also the fluorescence decay. Newport Green DCF ™ (NPG) is one such metal sensing complex developed for Zn sensing but also successfully used to detect Ni.

NPG is readily taken up by cells and distributed in the cytoplasm where metals such as Zn are expected to pool [23, 33]. In this work NPG was titrated with these two biologically relevant transition metals Ni and Zn and studied using optical spectroscopy. These in vitro studies showed that the addition of Ni caused the largest impact on NPG fluorescence intensity and dynamics.

Time-resolved fluorescence microscopy combined with confocal fluorescence microscopy was also performed on fixed mouse skin fibroblast cells (NIH 3 T3) preincubated at set extracellular concentrations of these transition metals in a time controlled manner. It was here observed that the modulation of the NPG fluorescence kinetics for Zn addition is in line with the in vitro observations. For Ni addition, on the other hand, the impact on the fluorescence kinetics is more complicated.

## Materials and Methods

**Fig. 1:**
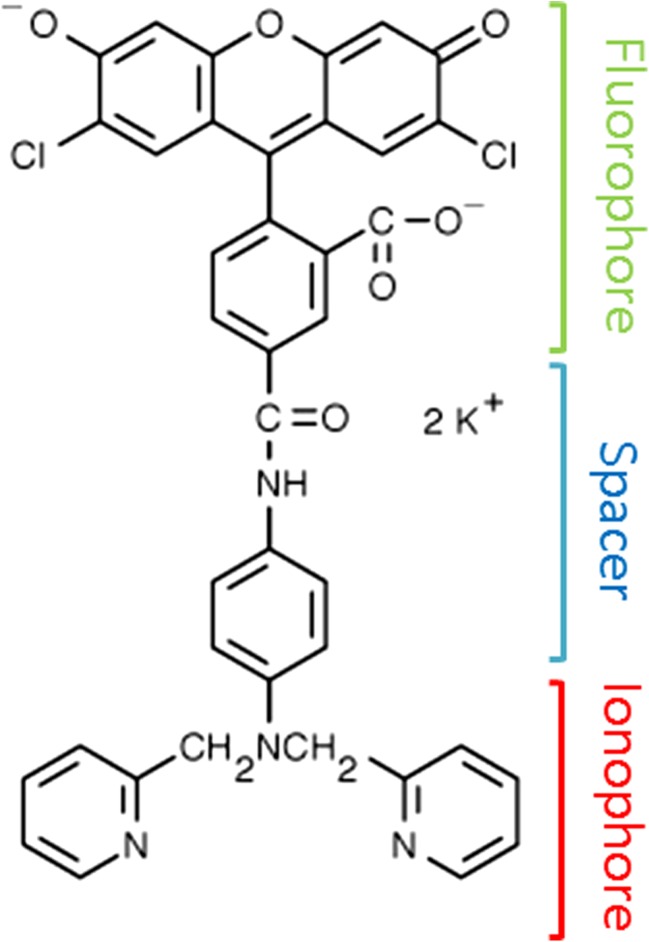
The molecular structure of the metal sensing complex Newport Green DCF ™ (NPG). See text for detail

Newport Green DCF ™ (NPG) was sourced from Life Technologies and used without further purifications or modifications (see Fig. 1). For spectroscopy measurements NPG was dissolved in a 25 mM Tricine (NaOH) buffer (pH = 8.0) unless otherwise stated. Fluorescein (Sigma Aldrich) was used without further purification and modification and was also prepared in 25 mM Tricine (NaOH) buffer (pH = 8.0).

Steady state optical absorption was performed using a Unicam UV-VIS ™ spectrophotometer and fluorescence using a JobinYvon Horiba Fluorolog ™.

Time-resolved fluorescence spectroscopy was performed using a PicoQuant pulsed diode laser LDH-P-C-485 (485 nm, 70 ps pulses FWHM @ 20 MHz). The emission was detected in a right angle geometry using suitable band pass/long pass filters (Comar Instruments) with a photon counting module Idquantic (id100–20) in combination with a Becker-Hickl SPC-130 time correlated single photon counting module. The data was subsequently fitted to a sum of exponentials;

3 $$ F(t)=\sum \limits_i{A}_i\exp \left(-{k}_it\right) $$

by deconvolution with the instrument response function (IRF) obtained from a light scattering solution of Ludox particles. The IRF has a full width half maximum (FWHM) of ~250 ps which provides a temporal time-resolution of ≥50 ps in the time-resolved fluorescence spectroscopy experiments.

Fluorescence microscopy was performed on fixed (4% PFA, Sapponin) NIH 3 T3 mouse skin fibroblast cells incubated in their recommended cell culture medium (DMEM/F12, 10% FBS pheol red free) containing 10 or 20 μM total NPG as reference. Cells were also pre-incubated using similar techniques as above with 5 *μ*M Zn or 50 *μ*M Ni containing buffered (25 mM HEPES) cell culture medium for 10 mins followed by PBS wash (×3) and incubation with 10 *μ*M NPG containing phenol red free culture media for 10 mins. Confocal fluorescence microscopy was subsequently performed using a Leica SP5 II system, using a 488 nm Ar^+^-ion laser for excitation (2 mW, 400 Hz/ line bidirectional scanning, emission range 500–550 nm). For point scanning time-resolved fluorescence microscopy; a home built system was used based on a Zeiss Axiovert 135 M Inverted Epi-fluorescence microscope. The excitation source was the PicoQuant diode laser LDH-P-C-485 previously described. A Zeiss 100x/1.4 N.A. oil immersion lens was used for the detection of the fluorescence selected using a 540 ± 25 nm band pass filter (Comar Instruments). The data was subsequently fitted to a sum of exponentials. The IRF in the time-resolved fluorescence microscopy was obtained through light scattering from Ludox particles dispersed on a microscope slide. The FWHM was ~500 ps which afford a temporal time-resolution of ≥100 ps.

## Results and Discussion

**Fig. 2:**
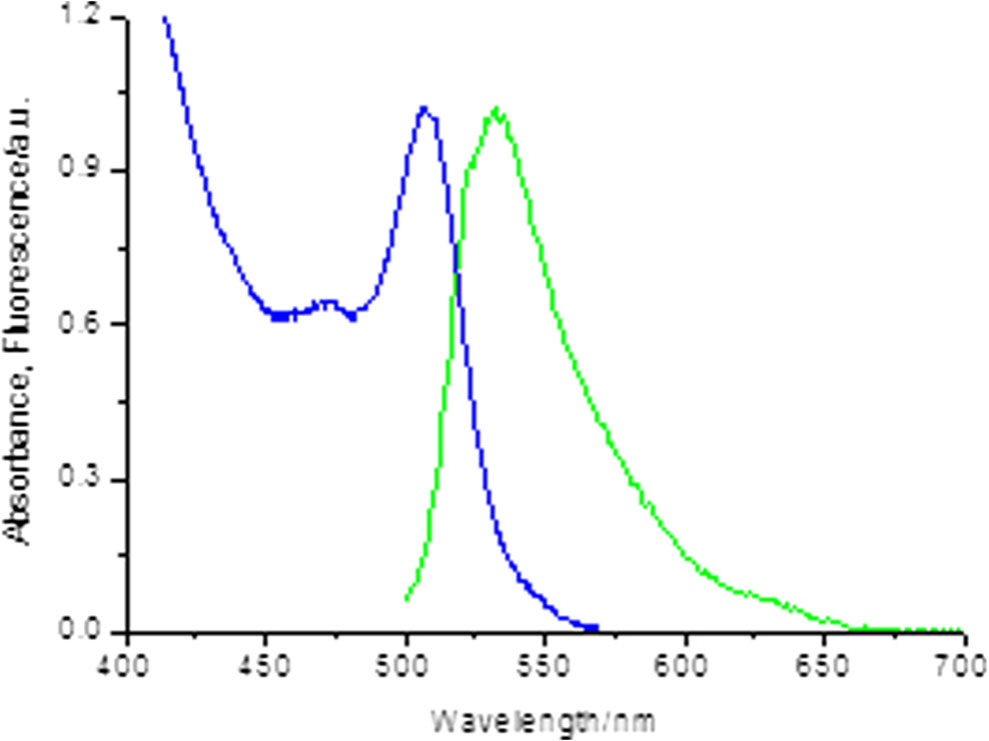
Normalised absorbance and fluorescence of NPG in Tricine buffer excited at 485 nm. See text for details

As NPG contains a fluorescein derivative as its signalling moiety, it is informative to compare and contrast the photo physics of NPG and fluorescein under similar conditions. NPG in tricine buffer displays absorption and emission maxima at 505 nm and 535 nm respectively (Fig. 2). This gives a Stokes shift of 1100 cm^−1^ while fluorescein (absorption maximum at 490 nm and fluorescence maximum at 515 nm, see SI) display a Stokes shift of 990 cm^−1^ thus showing almost the same degree of structural relaxation. The fluorescein fluorescence is sensitive to the environment and for its dianion derivative, which is the relevant form in this comparison, a dramatic redshift of absorption and fluorescence is observed in anhydrous solvents [34, 35]. It is, however, not an environmental effect that causes the bathochromic shift of the absorption and fluorescence in NPG, but rather a conjugation effect due to the covalently attached spacer and ionophore.

While the QY for the fluorescein dianion is well established (0.9 in NaOH, pH = 8.0), the QY for NPG has in this work been estimated to be 0.40 ± 0.02 in the absence of additional metals (Zn and/or Ni) using both the comparative method with fluorescein/NaOH (at pH = 8.0) as the reference and absolute method using an integrating sphere [36, 37]. As the Stokes’ shift of the two systems is very similar, as previously mentioned, the lower QY for NPG is attributed to quenching by PET rather than larger rates of intra-molecular non-radiative decay processes summarised by the term∑*k*_nr_.

As data on pH dependence is scarce regarding the optical and electronic properties of NPG, a pre prepared (10^7^ cells / ml phenol red free cell media) NIH 3T3 cell lysate with 10 μM NPG where studied using absorption and fluorescence spectroscopy as the function of incremental pH adjustment The rational for undertaking these experiments was to explore the optical properties of NPG in the presence of the intra-cellular matrix. In this wide pH range it would not be reasonable to assume that normal organelle dependent cellular function lies within these studied pH values. A significant impact on the fluorescence intensity using an isobestic point in the spectrum as the reference is observed (see SI). NIH 3T3 cell lysate solutions at similar pH but with the presence of added Zn (10 μM) were examined in parallel where a significant intensity increase was observed following a similar, but somewhat slightly shifted pH dependent trend. The absorption was also monitored in conjunction with the fluorescence (for NPG in NIH 3T3 cell lysate, with and without Zn present) displaying a parallel pH dependent absorption increase (see SI). This implies that the observed fluorescence intensity variations are not necessarily due to a modulation of the QY but rather caused by an environmentally induced photo physical effect on the absorption and emission properties on chromophore of the NPG complex.

**Fig. 3:**
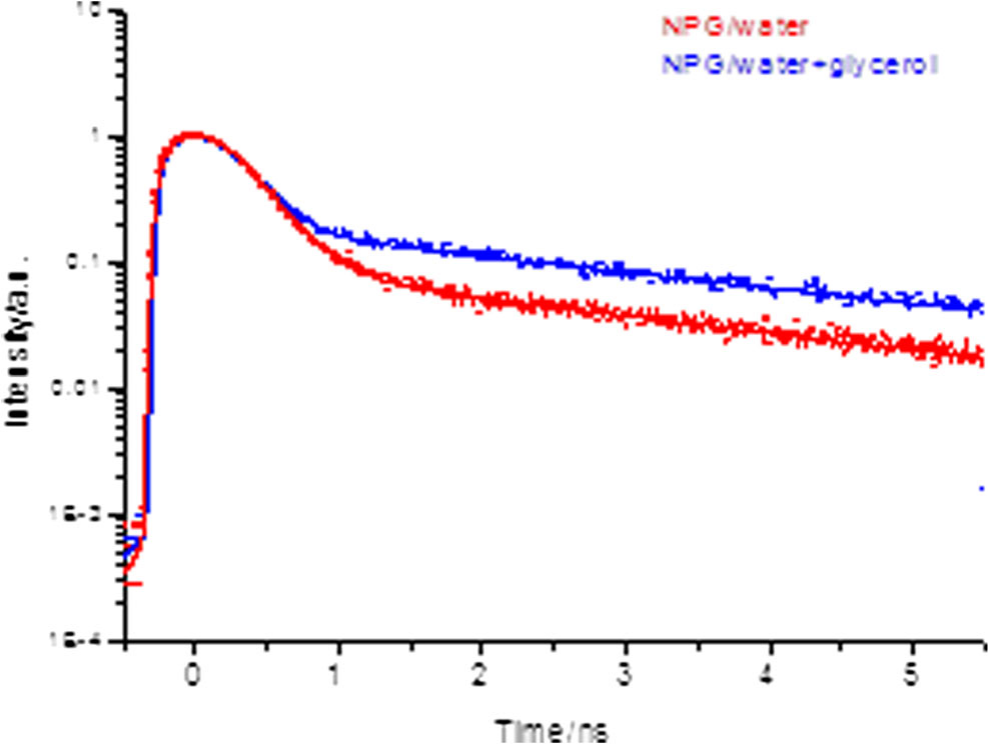
Normalised time-resolved fluorescence of NPG in water and water/glycerol excited at 485 nm. Dots are raw data and solid line the fitted functions See Table 1 for data and text for details

The time-resolved fluorescence kinetics of NPG follows a bi-exponential decay across the entire fluorescence band. The underlying reason for this is the dynamics of the parallel PET process which is reflected in the fluorescence decay and which is distinct from the normal unperturbed fluorescence. To explore if this decay is due to a conformational change within the complex, NPG was also dissolved in a buffer + glycerol mixture (50:50) which should affect the extent of conformational change. However, as seen both in Fig. 3 and from the data in Table 1, the yield of the fast component *τ*_1_ increases in the more viscous media. This rules out conformational change having an impact on the fluorescence decay, instead the likely reason for the modulation of the fast component is more likely to be charge transfer and/or hydrogen bonding effects between solvent and solute in the buffer + glycerol mixture.

**Table 1:** Time-resolved fluorescence data for NPG in various environments and for addition of metals. Time-resolved fluorescence data for Fluorescein in buffer and for addition of metals. See text for details

System	χ^2^	τ_1_ (ns)	f_1_ (%)	τ_2_ (ns)	f_2_ (%)	τ_3_ (ns)	f_3_ (%)	< τ> (ns)
NPG/water	1.15	0.15	62	3.50	38	–	–	3.28
NPG/water + Glycerol	1.10	0.19	42	3.20	58	–	–	3.08
NPG/buffer	1.51	0.25	74	2.30	26	–	–	1.81
NPG/buffer +Zn	1.01	0.15	42	3.20	58	–	–	3.10
NPG/buffer + Ni	1.56	0.23	38	3.30	62	–	–	3.17
NIH 3T3 cell lysate/NPG pH = 4.05	1.03	0.06	3	0.41	22	2.1	75	2.01
NIH 3T3 cell lysate/NPG + Zn pH = 4.0	1.50	0.21	10	0.89	21	3.0	69	2.80
NIH 3T3 cell lystate/NPG pH = 4.90	1.06	0.09	3	0.46	31	2.4	66	2.24
NIH 3T3 cell lysate/NPG + Zn pH = 4.85	1.27	0.03	2	0.46	13	3.1	85	3.04
NIH 3T3 cell lysate/NPG pH = 5.35	1.09	0.09	4	0.48	25	2.5	71	2.37
NIH 3T3 cell lysate/NPG + Zn pH = 5.20	1.41	0.03	2	0.45	9	3.2	89	3.16
NIH 3T3 cell lysate/NPG pH = 6.00	1.01	0.05	3	0.44	10	2.8	87	2.76
NIH 3T3 cell lysate/NPG + Zn pH = 6.00	1.51	0.05	2	0.46	6	3.2	92	3.17
Fluorescein/buffer	1.14	4.47	100	–	–	–	–	4.47
Fluorescein/buffer + Zn	1.48	4.40	100	–	–	–	–	4.47
Fluorescein/buffer + Ni	1.51	4.44	100	–	–	–	–	4.47

**Fig. 4:**
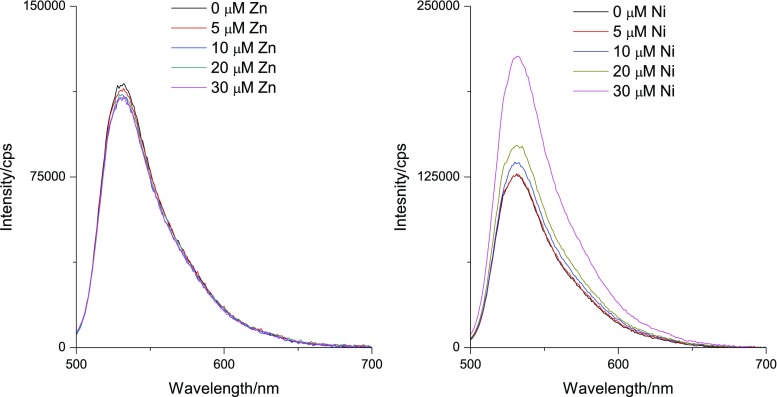
(Left) Fluorescence spectra of 5 μM Newport Green in 1 ml Tricine NaOH (25 mM) excited at 480 nm (five scan avg.), upon addition of Zn. (Right) Fluorescence spectra of 5 μM Newport Green in 1 ml Tricine NaOH (25 mM) excited at 480 nm, (five scan avg.) upon addition of Ni. See text for details

**Fig. 5:**
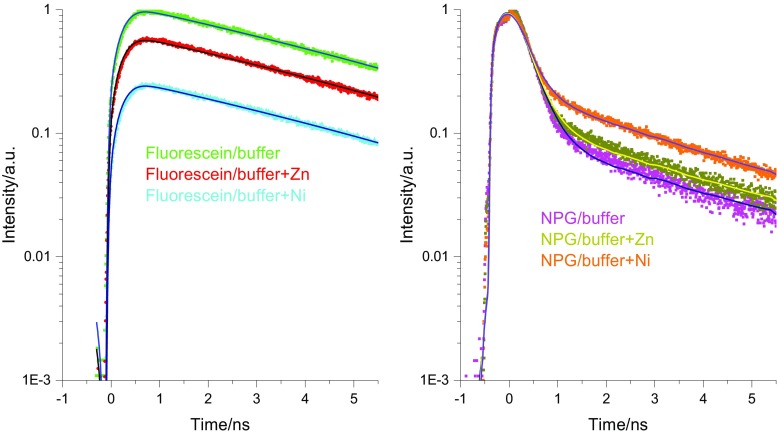
(Left) Time-resolved fluorescence for fluorescein in the absence and presence of Ni (1 mM) and Zn (1 mM) respectively in 1 ml Tricine NaOH (25 mM). Dots are raw data and solid line the fitted function. The different curves and fits have been separated by a scaling factor for clarity. (Right) Time-resolved fluorescence of 5 μl Newport Green in 1 ml Tricine NaOH (25 mM) with addition of Ni (1 mM) and Zn (1 mM). Dots are raw data and solid line the fitted function See Table 1 for data and text for details

To study the impact of metal ions present on the fluorescence, in vitro titrations were performed. Fig. 4 shows the steady state fluorescence spectra recorded at various concentration of Ni and Zn added to a NPG/tricine solution. For NPG in the presence of Zn the effect on the fluorescence is modest, while Ni addition leads to a clear intensity enhancement. We note the increase in intensity which confirms NPG is interacting with Ni leading to an impact on the internal PET within NPG. Other works have revealed a different scenario in which there is electronic energy transfer between organic chromophores and Ni [38, 39], this is clearly not the case here. In the time-resolved studies a similar trend emerges with a modest impact for Zn introduction while Ni addition resulted in an enhancement of the yield of the fast component, *τ*_1_ (see Fig. 5 and Table 1).

The pH dependence on the time-resolved fluorescence was studied in conjunction with the steady state fluorescence previously discussed, using NIH 3 T3 cells lysate bearing NPG, without and with additional Zn present in solution). These measurements provide more insight into excited state processes as they are independent of absorption cross-section and integrated fluorescence intensities. The data shows that at low pH < 4.5 and high pH > 6.0, Zn addition does not affect the fluorescence decay; the relative contributions of the two components in the bi-exponential decay are the same. However, in the range 4.5 ≤ pH ≤ 6.0, quenching of the fast decay phase is observed with addition of Zn.

**Fig. 6:**
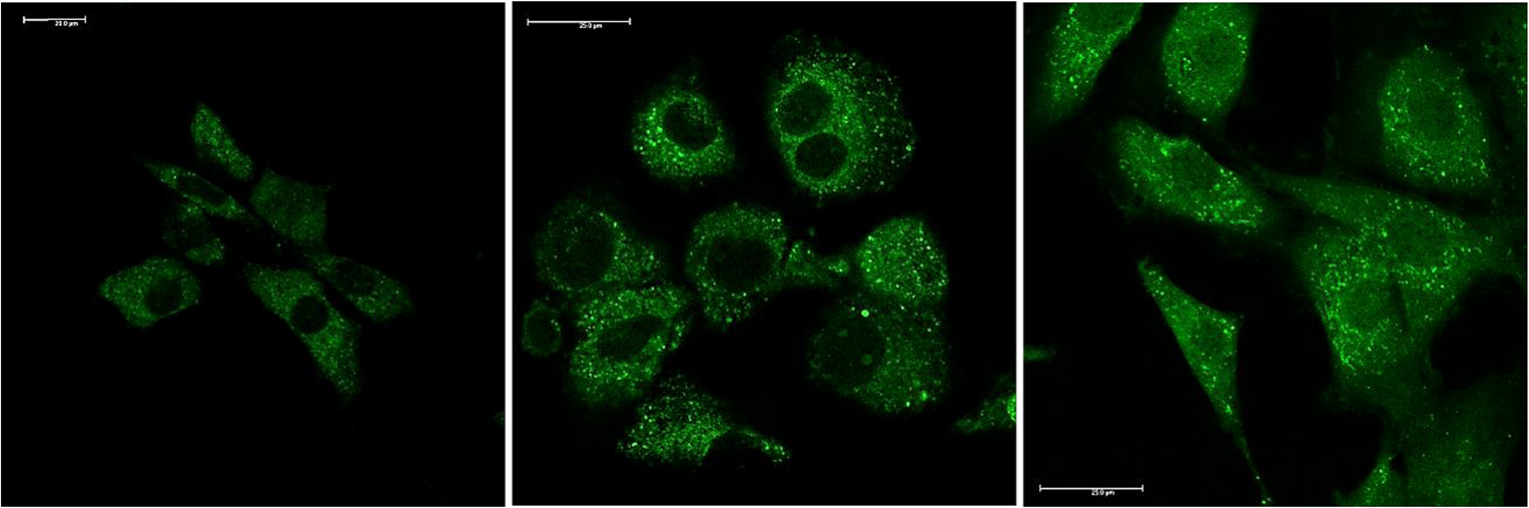
(Left) Confocal fluorescence image of NPG [20 μM] without added metals. (Middle) Confocal fluorescence image of NPG [10 μM] with added Zn [50 μM]. (Right) Confocal fluorescence image of NPG [10 μM] with added Ni [50 μM]. See text for details

The mechanism of metal sensing in NPG and other related systems are due to a well characterised PET process involving both the fluorophore and the ionophore (See Fig. 1). Photo excitation leads to an electron vacancy in the HOMO level of the fluorophore. In the absence of metals the HOMO of the ionophore is on a higher energy level relative to the fluorophore HOMO and the electron rich di-2-picoamine (ionophore) moiety can donate an electron to the fluorophore’s HOMO level. The consequence is a quenching of the fluorophore fluorescence as the LUMO level must donate its electron in a secondary electron transfer process. Complexation of a metal to the ionophore lowers its HOMO level and the electron transfer process to the fluorophore’s HOMO level is in this case more unfavourable. The consequence of this is more intense fluorophore fluorescence as this corresponding decay channel is favoured. While in this work an increase in fluorescence intensity is observed, in particular for Ni addition (see Fig. 4), the decay of the fluorescence remains bi-exponential. This indicates that PET is still influencing the fluorophore excited state decay although the amplitude of the fast decay component *τ*_1_ is decreasing with metal addition (See Fig. 6 and Table 1). By contrast, the data in Table 1 also shows that this component has a relatively large yield (> 60%) in the absence of metals. This clearly shows that the fast component is a reflection of fluorescence quenching due to electron transfer, the extent of which subsequently is reduced upon metal complexation to NPG.

To firmly establish that the modulation of the fluorescence originates from a PET process involving the sensing moiety of NPG and the added cations Ni and Zn, control experiments on fluorescein were performed. Henceforth, both steady state and time-resolved fluorescence were recorded with added Zn or Ni (1 mM respectively) to a fluorescein/tricine solution. This did not have any impact on either spectral form and spectral intensity or the kinetics. (See Fig. 6 and Table 1 for data. Additional data can also be found in the SI). It should also be noted that in fluorescein the presence and possible interactions with metals does not lead to intersystem crossing through a heavy atom effect.

Also previous works indicates more favorable Ni association to NPG as compared to Zn and NPG [40, 41]. The dissociation constant is given by the ratio;

4 $$ {K}_{\mathrm{d}}=\frac{{\left[{M}^{2+}\right]}^x{\left[ NPG\right]}^y}{\left[{M}_x^{2+}: NP{G}_y\right]} $$

where [*M*^2+^] is the concentration of Ni or Zn, and these are reported to be on the order of* K*_d_ ≤ 1 × 10^−6^ M for Ni while typical values are *K*_d_ ≈ 1 × 10^−5^ → 1 × 10^−6 ^M for Zn [40, 41]. The Pauling electron negativities for these two transition metals also imply that Ni would interact more strongly as compared to Zn; 1.91 eV for Ni compared to 1.65 eV for Zn. This is consistent with the results reported here; a stronger modulation of the fluorescence with addition of Ni to the NPG complex. However, it is slightly contradictory to the Irving-Williams series which stipulates strong complex formation for Zn as compared Ni [42].

Previous works has shown a large impact on the fluorescence lifetime in particular to Zn addition but also for Cu [36]. These works showed the potential of using NPG and related systems for metal sensing and crucially, to employ time-resolved fluorescence techniques in this context [43]. Frequency modulation techniques produce the average fluorescence lifetimes, generally given by the relation;

5 $$ \left\langle {\tau}_{\mathrm{f}}\right\rangle =\frac{\sum \limits_i{a}_i{\tau}_i^2}{\sum \limits_i{a}_i{\tau}_i}. $$

While this technique has it merits, such as a wide dynamic range, the detailed information of non-exponentially decaying fluorescence is not readily accessible.

Using the frequency modulation technique Thompson et al. observed a strong impact on the average fluorescence lifetime upon an increase in Zn addition; 〈*τ*_f_〉 = 0.88 ns to 〈*τ*_f_〉 = 2.93 ns when going from a low to high Zn concentration [44]. While conditions were slightly different compared to this work (modestly higher pH, different concentrations used) invoking the average lifetime analysis of the data obtained in this study did reproduce the same trends (See Table 1).

As mentioned in the introduction, some transition metals play key roles in the metabolism of bio-active systems. Although, labile free metal concentrations are low, elevated *in-cellulo* levels can cause disruption to cellular function. Loading fixed NIH 3T3 cells with NPG results in a predominantly lysosomal localisation of probe complexes, as shown in confocal fluorescence microscopy images of Fig. 6 (additional images in the SI). This localization profile is expected for this class of optical probes and is in line with what has been observed and previously reported [45] for similar systems such as fluorescein derivatives. This suggested localization profile has also been verified using the commercial stain LysoTracker Red (images not shown.) with colocalizaton coefficients in the region of *P* > 0.60. This predominantly lysosomal localization profile has prompted us to suggest that the *in-cellulo* measured photophysical parameters, of NPG, and its metal adducts where studied, are indeed a blend of emissive species at different pH. The localization profile, despite been verified to be predominantly lysosomal also display clear characteristics of sensor accumulation in the Mitochondiral network (costained with MitoTracker Red, not shown), possibly covalently alkylating mitochondrial DNA and in the cytosol. This localization profile could be attributed to the fixed nature of the studied cells via various well-documented membrane permeablisitation mechanisms, and somewhat the observed localization in live-cells would be different. But as for reasons detailed above only fixed cells were studied. The pH of an unperturbed lysosome is in the range of 4.2–4.5, whilst the cytosol and the dynamically equilibrated mitochondrial network pH is close to 7.4–7.6 [46]. However it is worth also mentioning that based on absorbance and emission vs pH plots (SI), and due to low absorbance and subsequent sensor brightness at low lysosomal pH; in order for the NPG to be detectable in such bright spots in the lysosome orders of magnitude higher local sensor concentration is expected in the lysososme. Another possibility for this bright lysososmal staining could be the result of elevated lysosomal pH upon NPG localization. However as the natural dynamic homeostatic nature of the cell has been shut down due to fixing this is highly unlikely.

Point scanning of the time-resolved fluorescence of similar clusters of cells as shown in Fig. 6, was performed on a number of areas on each microscope slide as a mean to obtain a statistically robust result. It was observed that also for compartmentalization in cells, the time-resolved fluorescence of NPG is bi-exponential. The decay is dominated by the fast component *τ*_1_ which has a yield of ~60%. Cells were exposed to concentration of NPG of 10 mM and 20 mM in the growth medium. The later concentration led to a higher uptake of NPG and consequently more intense fluorescence and brighter images but crucially, without any impact on the fluorescence decay (See Table 2). This finding suggests that NPG is not sensing any of the relevant metals under what must considered as normal conditions for the cell. However, this is not an unexpected result as the concentrations of in particular labile Zn is expected to in the range ~10^–9 M^ [24]. This would be 2–3 orders of magnitudes below the detection limit of NPG, as previously discussed.

**Table 2:** Time-resolved fluorescence microscopy data for NPG loaded NIH 3T3 cells with addition of metals. The data in this table is based on a surveying a number of different samples and different areas of interest within each samples. For Ni [50 μM] there is a large statistical spread of f_1_ which is indicated by the range. See text for details

System	Metal	τ_1_ (ns)	f_1_ (%)	τ_2_ (ns)	f_2_ (%)	< τ> (ns)
NPG [10 μM]	–	0.20 ± 0.10	62	1.20 ± 0.10	38	0.98
NPG [20 μM]	–	0.40 ± 0.10	58	1.35 ± 0.10	42	1.07
NPG [5 μM]	Zn [5 μM]	0.22 ± 0.05	22	2.30 ± 0.10	78	2.24
NPG [10 μM]	Zn [50 μM]	0.37 ± 0.05	42	1.40 ± 0.20	58	1.42
NPG [10 μM]	Ni [50 μM]	0.49 ± 0.05	38–55	1.60 ± 0.10	62–45	1.29

**Fig. 7:**
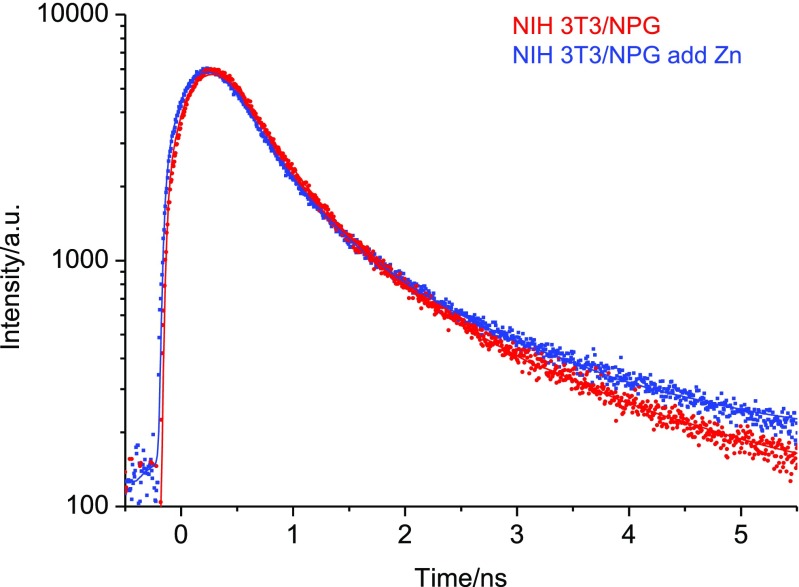
Time-resolved fluorescence microscopy from a point scanning experiment on fixed NIH 3T3 cells with NPG and added Zn. The two decays are normalized to the same intensity at *t* = 0. For the NIH 3T3/NPG decay χ^2^ = 1.51 while for NIH 3T3/NPG add Zn decay χ^2^ = 1.42 See Table 2 for data and text for details

The second fluorescence decay phase (*τ*_2_) is in general shorter in vivo as compared to the in vitro measurements in the tricine buffer as can be seen when comparing data in Table 2 with Table 1. It is known that the pH in the lysosmal regions of the cell, is 4.2–4.5 which differs from the cell media and cytosol pH. This has an impact on the chromophore of NPG which is then reflected in the fluorescence decay. The data obtained on cell lysate at different pH (with and without metal addition) suggest that the excited state kinetics of NPG is affected with an increase in the fluorescence rate (lifetime decrease) at low pH. The implication is that also the QY is affected (lowered) in parallel. The introduction of additional Zn, to the growth medium, regardless of simultaneous, post- or pre-NPG incubation, led to cellular uptake and modulation of the fluorescence decay, accordingly. In accordance with the in vitro measurements it is observed that the yield of the fast component *τ*_1_ drops to 35–40%. This effect is clearly seen in Fig. 7. The implication is therefore that; i) the Zn co-localises with NPG in the cytoplasm and more importantly mainly localizing in the lysosomes of the cell, ii) NPG can sense the elevated intracellular Zn concentration which likely will be in the ~10^−6^ M range. Also in this experiment, the added metal concentrations were varied slightly but the fluorescence decay analysis returned similar results (See Table 2) indicating strong 1 to 1 binding.

Addition of Ni to the growth medium with a view to facilitate free cellular uptake on fixed cells was also exploited. It should be recognized, as noted in the introduction that Ni is not a naturally occurring transition metal in mammalian cells. Uptake and subsequent localization could therefore be slightly different, as compared to Zn. However, confocal fluorescence images of cells with additional Ni introduced, shows no difference in localisation profile of NPG. This indicates that either the fluorophore or ionophore moiety determines the mechanism of uptake and cellular localization possibly via protein mediated uptake or shuttling to selected organelles or by simple concentration gradient diffusion through permeated cellular membranes. Live cell imaging has not been executed due to the possible, previously detailed, toxic nature of Ni.

The time-resolved fluorescence monitored in point scanning as previously described, shows a more complicated picture. In contrast to the situation with excess Zn load, which gave a clear impact on the yield of *τ*_1_, there is a more varied response when Ni is exposed to mammalian cells. The results from a number of different samples show that the *τ*_1_ yield has a substantially larger variation. Although the confocal fluorescence images show similar localization profiles for NPG with Ni exposure the time-resolved fluorescence microscopy implies that Ni is not detected consistently. There can be two possible explanations for this; i) Ni uptake is low or alternatively Ni uptake is effective but retention in the cytoplasm is high with very little being internalized in the lysosomes, ii) Ni migrates into regions of the cell beyond the detection of NPG. This would suggest accumulation of Ni in the nuclei. The later scenario is not unreasonable as Ni considered being a toxic and carcinogenic compound when accumulated in mammalian cells.

With regard to Ni sensing a recent work on sensing Ni in photosynthetic cyanobacteria is relevant in this context [47]. NPG was successfully used and with elevated Ni concentrations the time-resolved fluorescence microscopy showed a single exponential decay of the fluorescence with a 2.4 ns lifetime. This suggest a very efficient Ni complexation to NPG and as a consequence complete inhibition of the intramolecular PET process. One should recognize the differences between the mammalian cells used in this work and the photosynthetic cyanobacteria (size, intracellular environments etc) but the same trends are observed through the application of time-resolved fluorescence microscopy.

## Conclusions

The use of time-resolved fluorescence as a signaling tool in metal sensing is demonstrated in this work. In vitro characterisation of the of the Ni and Zn sensing complex NPG shows that a fast phase of the bi-exponential fluorescence decay is modulated upon addition of Ni or Zn. *In cellulo* sensing in NIH 3T3 cells loaded with additional Zn reproduces the same trends in the decay of the fluorescence, for Ni addition the outcome is more complicated as previously discussed. Crucially, the changes in revealed by the time-resolved fluorescence microscopy are too subtle to be observed by intensity variations in conventional confocal fluorescence microscopy. This work therefore demonstrates the methodology of microscopic sensing and this has the potential to become an important method for further and more detailed studies of intracellular function in bio-active systems. A key aspect in this pursuit is to ensure the best possible temporal time-resolution is available, which in turn will require a reconvulution of the fluorescence decay with an IRF function. As shown in this work, this time-resolution is required as modulation of the fluorescence in organic fluorophores will result in fluorescence decays with a rate limit on the 100 ps time scale.

## Electronic supplementary material

ESM 1 (DOCX 4528 kb)
