# Supplementary material for: In Vitro and in Cellulo Sensing of Transition Metals Using Time-Resolved Fluorescence Spectroscopy and Microscopy
Source: J Fluoresc. 2018 Dec 26;29(1):255–63. doi: 10.1007/s10895-018-2335-z (PMC6428955; doi:10.1007/s10895-018-2335-z)
Supplement: Supplementary file 1 — (DOCX 4528 kb) [file 10895_2018_2335_MOESM1_ESM.docx]

**Supplementary information**

**In vitro and in cellulo sensing of transition metals using time-resolved fluorescence spectroscopy and microscopy**

**Robert Pal, Abigail C.J. Barker, Daniel Hummel and Lars-Olof Pålsson*.**

Department of Chemistry, Durham University, Lower Mountjoy, Stockton Road, DH1 3LE Durham United Kingdom.

Email: [lars-olof.palsson@durham.ac.uk](mailto:lars-olof.palsson@durham.ac.uk)

**Figure SI - 1.** Normalised absorption (solid line) and fluorescence emission (dashed line) of Fluorescein in 25 mM Tricine (NaOH) buffer.

**1 - T**

**Figure SI - 2.** An overlay of the photo luminescence excitation (at 600 nm) (solid line) and 1 - T spectra (dashed line) of 8 μl Newport Green DCF in 1 ml, 25 mM Tricine (NaOH) buffer.

**Figure SI - 3.** Ratio of fluorescence intensities as a function of pH (red squares) at 548 nm to 511 nm of Newport Green DCF in NIH 3T3 cell lysate, for excitation at 488 nm. Ratio of absorption strength as a function of pH (blue circles) at 516 nm to 446 nm.

**Figure SI - 4.** Ratio of fluorescence intensities as a function of pH with 5 μM Zn addition (red squares) at 548 nm to 511 nm of Newport Green DCF in NIH 3T3 cell lysate, for excitation at 488 nm. Ratio of absorption strength as a function of pH (blue circles) at 516 nm to 446 nm.

**Figure SI-5**. Intensity variation of NPG fluorescence as a function of the pH in NIH 3T3 cell lysate (red squares) and with 5 uM added total Zn (blue dots). See text for details.

**Figure SI - 6.** Emission spectra of fluorescein in 1 ml NaOH (1 mM). Excitation wavelength 475 nm, three scans averaged. For the same fluorescein stock solution; 10 μl of 1 mM ZnCl2 and 10 μl of 1 mM NiSO4 were added and emissions were measured again the same conditions**.**

**Figure SI - 7.** Time-resolved fluorescence decays of fluorescein in 1 ml NaOH (1 mM) for detection at 540 ± 15 nm and excitation at 485 nm. For the same fluorescein stock solution; 10 μl of 1 mM ZnCl2 and 10 μl of 1 mM NiSO4 were added and emissions were measured again the same conditions**.**


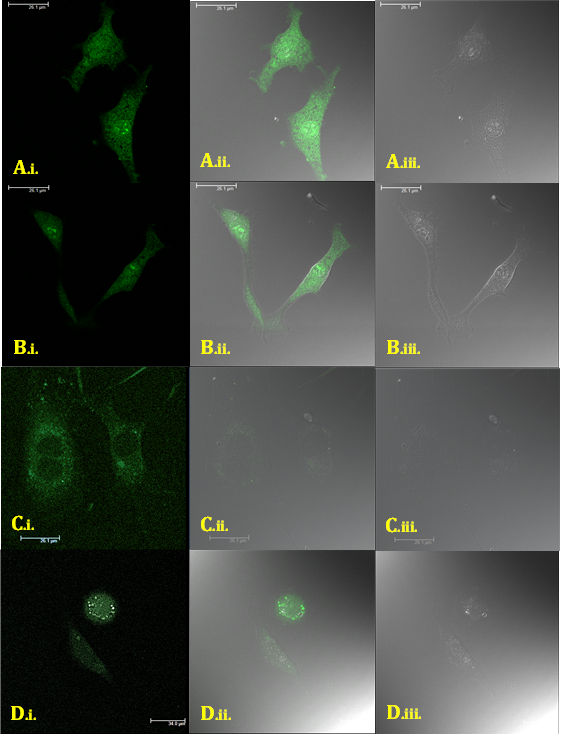


**Figure SI – 8.** Confocal microscope images of NIH-3T3 cells doped with Newport Green DCF. A and B are cells loaded with 5 µM NPG and 5 µM Nickel, C with 5 µM NPG no additional metal load and D with 10 µM NPG no metal present. Scale bar = 26.1 µm.
